# Supraspinal and Peripheral, but Not Intrathecal, σ_1_R Blockade by S1RA Enhances Morphine Antinociception

**DOI:** 10.3389/fphar.2019.00422

**Published:** 2019-04-24

**Authors:** Alba Vidal-Torres, Begoña Fernández-Pastor, Alicia Carceller, José Miguel Vela, Manuel Merlos, Daniel Zamanillo

**Affiliations:** Drug Discovery and Preclinical Development, Esteve Pharmaceuticals, Parc Científic Barcelona, Barcelona, Spain

**Keywords:** sigma-1 receptor, S1RA, morphine, nociceptive pain, antinociceptive effect, concentric microdialysis

## Abstract

Sigma-1 receptor (σ_1_R) antagonism increases the effects of morphine on acute nociceptive pain. S1RA (E-52862) is a selective σ_1_R antagonist widely used to study the role of σ_1_Rs. S1RA alone exerted antinociceptive effect in the formalin test in rats and increased noradrenaline levels in the spinal cord, thus accounting for its antinociceptive effect. Conversely, while systemic S1RA failed to elicit antinociceptive effect by itself in the tail-flick test in mice, it did potentiate the antinociceptive effect of opioids in this acute pain model. The present study aimed to investigate the site of action and the involvement of spinal noradrenaline on the potentiation of opioid antinociception by S1RA on acute thermal nociception using the tail-flick test in rats. Local administration was performed after intrathecal catheterization or intracerebroventricular and rostroventral medullar (RVM) cannulae implantation. Noradrenaline levels in the spinal cord were evaluated using the concentric microdialysis technique in awake, freely-moving rats. Systemic or supraspinal administration of S1RA alone, while having no effect on antinociception, enhanced the effect of morphine in rats. However, spinal S1RA administration did not potentiate the antinociceptive effect of morphine. Additionally, the peripherally restricted opioid agonist loperamide was devoid of antinociceptive effect but produced antinociception when combined with S1RA. Neurochemical studies revealed that noradrenaline levels in the dorsal horn of the spinal cord were not increased at doses exerting potentiation of the antinociceptive effect of the opioid. In conclusion, the site of action of σ_1_R for opioid modulation on acute thermal nociception is located at the peripheral and supraspinal levels, and the opioid-potentiating effect is independent of the spinal noradrenaline increase produced by S1RA.

## Introduction

The sigma-1 receptor (σ_1_R) has been described as the first ligand-regulated molecular chaperone located at the endoplasmic reticulum and plasma membranes whose activity is regulated in an agonist-antagonist manner. The σ_1_R is expressed in key areas for pain control and there is cumulative evidence supporting an involvement of the σ_1_R mainly in two kinds of pain conditions: (1) those involving sensitization, e.g., after sensitization with capsaicin or formalin or following nerve injury where σ_1_R antagonists by themselves inhibit pain behaviors in the absence of opioids (Romero et al., 2012; Vidal-Torres et al., 2014; Gris et al., 2016); and (2) in acute pain conditions after the application of mechanical (paw pressure test) or thermal (tail-flick and hot plate tests) nociceptive stimuli, where σ_1_R antagonists by themselves fail to modify the nociceptive thresholds but enhance opioid-induced antinociception (Sánchez-Fernández et al., 2013; Vidal-Torres et al., 2013; Merlos et al., 2017; Sánchez-Fernández et al., 2017).

S1RA (also known as MR309 or E-52862) is a σ_1_R antagonist with high affinity for σ_1_R, good σ_1_/σ_2_ selectivity ratio (>550), and selectivity against a panel of 170 receptors, enzymes, transporters, and ion channels (Romero et al., 2012). We previously reported that co-administration of S1RA with several opioids used in clinics results in an enhancement of the antinociception but not of undesired opioid-induced phenomena such as the development of analgesic tolerance, physical dependence, or inhibition of gastrointestinal transit. Moreover, S1RA restored morphine antinociception in tolerant mice and reversed the reward effects of morphine (Vidal-Torres et al., 2013). S1RA has been recently developed as a first-in-class analgesic drug. It has shown good safety and tolerability profiles after single and multiple doses in healthy humans in phase I clinical trials (Abadias et al., 2013); S1RA has also shown promising results in phase II clinical trial for neuropathic pain (Bruna et al., 2018).

Regarding the site of action, recent studies demonstrated that S1RA exerts by itself an antinociceptive effect after spinal, supraspinal and peripheral administration in the formalin-induced pain model in rats (Vidal-Torres et al., 2014), and also after peripheral administration in carrageenan-induced pain models in mice (Gris et al., 2014; Tejada et al., 2014; Gris et al., 2015). Recent information advocates that modulation by σ_1_R ligands of opioid antinociception occurs at the peripheral level, as shown in the paw pressure mechanical acute model (Sánchez-Fernández et al., 2014; Tejada et al., 2018). In contrast, available information also shows that this modulation on opioid antinociception occurs at the supraspinal level in the acute thermal nociception test in mice (Mei and Pasternak, 2002; Mei and Pasternak, 2007).

At the neurochemical level, S1RA increased noradrenaline (NA) levels in the dorsal horn of the spinal cord after intraplantar injection of formalin. Accordingly, intrathecal pre-treatment with the selective α_2_-adrenoceptor (α_2_-AR) antagonist idazoxan blocked the antinociceptive effect of S1RA (Vidal-Torres et al., 2014). No studies addressing this issue are available in relation to the opioid potentiating effect.

To gain further insight into the mechanisms underlying the modulatory effect of σ_1_R on opioid antinociception, we selected S1RA as a tool compound because it is one of the most characterized selective σ_1_R antagonists and the only one that has been evaluated in clinical trials with an intended indication for pain relief. S1RA efficacy in combination with morphine was studied by using different routes of administration in the tail-flick acute thermal nociceptive pain model in rats. The possible involvement of spinal NA in the potentiating effect was also investigated by using the concentric microdialysis technique in awake, freely-moving rats.

## Materials and Methods

### Animals

All animal husbandry and experimental procedures complied with the European guidelines for the protection of animals used for experimental and other scientific purposes (Council Directive of 22 September 2010, 2010/63/EU), and were approved by the local Ethics Committee. The results are reported in accordance with the ARRIVE guidelines for reporting experiments involving animals (McGrath et al., 2010). Male Wistar rats weighing 230–330 g (Charles River, France) were used. Naïve animals were housed in groups of four and housed individually after surgery. They had free access to food and water and were kept in controlled laboratory conditions with temperature at 21 ± 1°C and a light-dark cycle of 12 h (lights on at 7:00 a.m.). Experiments were carried out in a soundproof, air-regulated experimental room during the light phase. Each animal was used in a single experiment only.

### Drugs and Drug Administration

Morphine hydrochloride was obtained from the Spanish Drug Agency (Agencia Española de Medicamentos y Productos Sanitarios, Area Estupefacientes (Madrid, Spain)). Loperamide hydrochloride and naloxone-methiodide were obtained from Sigma-Aldrich. 4- (2- (5- methyl-1- (naphthalen-2- yl)- 1H-pyrazol-3- yloxy) ethyl) morpholine hydrochloride (S1RA; E-52862) (Díaz et al., 2012) was synthesized at Esteve Pharmaceuticals (Barcelona, Spain). Morphine (2.5, 5, and 10 mg/kg), naloxone-methiodide (4 mg/kg) and S1RA (10, 20, 40, and 80 mg/kg) were dissolved in 0.5% hydroxypropyl-methylcellulose (HPMC) (Sigma-Aldrich), and loperamide (1, 2, and 4 mg/kg) was dissolved in HPMC containing 0.5% Tween 80 and was administered intraperitoneally (i.p.) at 2 mL/kg. Naloxone-methiodide was administered 5 min prior to loperamide and S1RA. Baseline responses were always obtained prior to treatment administration. For intrathecal (i.t., volume: 10 μL), intracerebroventricular (i.c.v., volume: 10 μL bilaterally) and rostroventral medulla (RVM, volume: 1 μL) administrations, S1RA (80, 160, or 320 μg) was dissolved in cerebrospinal fluid (CSF, Perfusion Fluid CNS, CMA) and co-administered with systemic morphine (i.p., 2.5 or 5 mg/kg). I.t. and i.c.v. S1RA doses were selected based on a previous study where S1RA showed antinociceptive effects in the formalin-induced pain model (Vidal-Torres et al., 2014). Doses are expressed as the salt forms of the drugs.

### Antinociceptive Assay (Tail-Flick Test)

To evaluate the acute antinociceptive effects of the drugs and their combination, the nociceptive responses to acute thermal (heat) stimulation were assessed by the tail-flick test as previously described (D’Amour and Smith, 1941). Briefly, animals were gently restrained with a cloth to orient their tails toward the source of heat of the tail-flick apparatus (Panlab, Barcelona, Spain). A noxious beam of light was focused on the tail about 5 cm from the tip, and the tail-flick latency (TFL, latency to remove the tail as of the onset of the radiant heat stimulus) was recorded automatically to the nearest 0.1 s. The intensity of the radiant heat source was adjusted to yield baseline latencies between 2 and 4 s and a cut-off time was set at 10 s to avoid heat-related tail damage.

The effect of treatments on TFL was calculated by the formula %Antinociception = ((Individual test latency – Individual baseline latency)/(Cut-off latency – Individual baseline latency)) × 100. When appropriate, the ED_50_ value was estimated from the dose-response curve.

### Intrathecal Catheterization and Administration

Catheterization of the spinal subarachnoid space was conducted as previously described (Storkson et al., 1996; Pogatzki et al., 2000) with i.t. catheters (No. 0007740, Alzet) under anaesthesia with pentobarbital (i.p., 60 mg/kg, 2 mL/kg). The lower dorsal pelvic area corresponding to vertebral L3-S3 was shaved and prepared with povidone-iodine. A midline longitudinal skin incision was made (2–3 cm) and the space between the lumbar vertebrae L5 and L6 was punctured with a 22G hypodermic needle. Tail-flick or hind paw retraction indicated an i.t. position. A 28G PU catheter (10 cm length, 0.36 mm OD; 0.18 mm ID, Alzet) reinforced with a teflon-coated stainless steel stylet was advanced cranially 4 cm through the needle to reach the L4-L5 medullar area. The needle and the stylet were removed and the catheter was withdrawn so that 5 cm extended outside of the lumbar muscles. Superglue-3 gel (Loctite^®^) was used to fix the catheter to the fascia. The distal end of the 28G PU catheter was connected with super glue to an 8 cm tube (0.84 mm OD; 0.36 mm ID) ended with an Alzet connection (1.02 mm OD; 0.61 mm ID). The catheter was tunneled under the skin to the cervical region, flushed with CSF and sealed with a cautery pen. The skin was then closed and animals were allowed to recover in individual cages for 7 days. Catheterized rats had no detectable motor deficits. S1RA or CSF were injected i.t. with a 50 μL Hamilton syringe at a volume of 10 μL over a period of 20 s, followed by 20 μL of CSF to flush the catheter. At the end of the experiment, the animals were killed by CO_2_ inhalation, 10 μL of fast green was injected i.t., and the level and side position of the catheter tip were confirmed. Epidural catheterizations (15%) were discarded and only i.t. catheters were considered.

### Intracerebroventricular and RVM Cannulae Implantation and Administration

Bilateral i.c.v. administration guide cannulae (26 GA, 0.46 mm OD, 0.24 mm ID, 5 mm long, Plastics One) or a RVM administration guide cannulae (26 GA 20 mm, C315G/SPC, Plastics One) were stereotaxically implanted in rats anaesthetized with pentobarbital (i.p., 60 mg/kg, 2 mL/kg). With the incisor bar set at 0 or -5 mm, the coordinates from bregma were -0.8 AP, -1.6 L, and -3.5 DV; or -10.8 AP, 0.0 L, and -4.3 DV (from the dura mater) for i.c.v. and RVM, respectively. Stainless steel guide cannulae were secured to the skull with two anchor screws and dental acrylic. Animals were housed in individual cages, disinfected daily with povidone-iodine and allowed 6–7 days to recover from surgery. In RVM-implanted rats, 18 h prior to the test, after removing the dummy cannulae (Plastics One), an internal cannula (33 GA, C315IA/SP, Plastics One) extending 6 mm past the guide cannula was introduced under isoflurane anaesthesia. S1RA or CSF were injected i.c.v. with a 10 μL Hamilton syringe at a volume of 5 μL (per cannula) over a period of 20 s, followed by 1.8 μL of CSF to flush the cannula. S1RA or CSF were injected RVM with a 5 μL Hamilton syringe at a volume of 1 μL over a period of 20 s, followed by 1.8 μL of CSF to flush the cannula. After experimental testing, the animals were killed by CO_2_ inhalation and fast green was injected for cannula placement examination. Only animals with correct cannula placements (100% in i.c.v. implantation and 90% in RVM implantation) were included in data analyses.

### Microdialysis Surgical Procedures/Microdialysis Probe Implantation in Spinal Cord

Rats were anaesthetized with chloral hydrate (i.p., 440 mg/kg) and placed on a David Kopf stereotaxic frame. The dorsal zone corresponding to the thoracic vertebra (Th13) was shaved and prepared with povidone-iodine. An incision was made along the dorsal midline such that the muscle overlaying the Th13 and the first lumbar vertebra (L1) could be removed. Th13 was then immobilized on the horizontal plane by using a transverse process clamp and a burr hole was made in the dorsal surface. The exposed dura mater was then carefully opened and a microdialysis probe of concentric design (CMA/11) was inserted into the spinal cord at an angle of 45° from the vertical plane. The microdialysis probes (exposed tip 2.0 mm × 0.24 mm) were implanted into the medial DH of the L4 lumbar region of the spinal cord. The probe was fixed by applying superglue-3 gel (Loctite^®^) and dental cement around the probe and by a stainless steel anchorage screw located in the Th13 vertebra. The skin was then closed and rats were allowed to recover overnight, one per cage, with free access to food and water. Only implanted rats showing normal behavior after the recovery period (no walking dysfunction, normal weakened extension withdrawal reflex of the hind limb, no reduced toe spread, normal food and water intake, no piloerection or apparent stress signals) were considered in the study, and were used only once. At the end of the experiment, the animals were killed by CO_2_ inhalation and spinal cords were dissected out for histological examination to verify that microdialysis probes were correctly implanted. Only animals with correct probe placements (90%) were included in data analyses.

### Sample Collection in Awake Rats

Around 20 h after probe implantation, rats were placed individually in a system for freely moving animals. The dialysis probes were connected to a CMA microdialysis system, then perfused with CSF perfusion fluid at 1.5 μL min/1 flow rate, and consecutive samples were collected into vials every 15 min. The probe was perfused for 1 h for stabilization of baseline NA release. This was followed by a 90 min period for baseline sample collection. Animals received systemic (i.p.) morphine (5 and 10 mg/kg) or vehicle + systemic (i.p.) S1RA (40 and 80 mg/kg) or vehicle and were perfused for 180 min. Dialysis sampling was performed separately in groups of rats other than those used for tail-flick assessment of operated animals in order to avoid excessive rat handling likely to interfere with NA level determination.

### Analytical Procedures

Dialysate samples were assayed for NA content by reversed-phase high-performance liquid chromatography (HPLC) coupled with electrochemical detection. The mobile phase was 75 mM phosphate buffer (pH 6) containing 0.35 mM octanesulfonic acid and 0.2 mM ethylenediamine tetra-acetic acid (EDTA) with 25% of methanol. Separation was carried out with a Gemini C18 110A (3 μm) column, connected to a Waters 2465 electrochemical detector at 35°C and operated at a flow rate of 0.2 mL/min. Detection was performed by oxidation at 0.45 V. Values were not corrected for *in vitro* recovery through the dialysis probe.

### Statistical Analysis

Data were expressed as means ± S.E.M. The mean values of four dialysate samples obtained before treatment administration were considered as the 100% baseline values. The extracellular NA concentration of dialysate samples collected during an experiment were normalized as percentage of the baseline values. Treatment groups were compared with appropriate control groups using one-way or two-way ANOVA analysis of variance followed by the Newman-Keuls multiple comparison test or followed by the Bonferroni *post hoc* test, respectively, as appropriate. ED_50_ values were determined using a four-parameter logistic equation (sigmoidal dose-response curve, variable slope) with the top or bottom fixed (DeLean et al., 1978). The ED_50_ was defined as the dose that produced 50% of the maximum possible effect. Drug effects were expressed as area under the curve (AUC) within the same subject, as calculated using the linear trapezoidal method and were compared to vehicle using one-way ANOVA followed by Newman–Keuls multiple comparison test. In all cases the level of statistical significance was set at *P* < 0.05. ED_50_ values with 95% confidence intervals (CI), calculation of the area under the curve (AUC) and statistical analyses were computed using GraphPad Prism version 5 software (San Diego, CA, United States).

## Results

### Systemic S1RA Enhanced the Antinociceptive Effect of Systemic Morphine in the Tail-Flick Test in Rats

**FIGURE 1:**
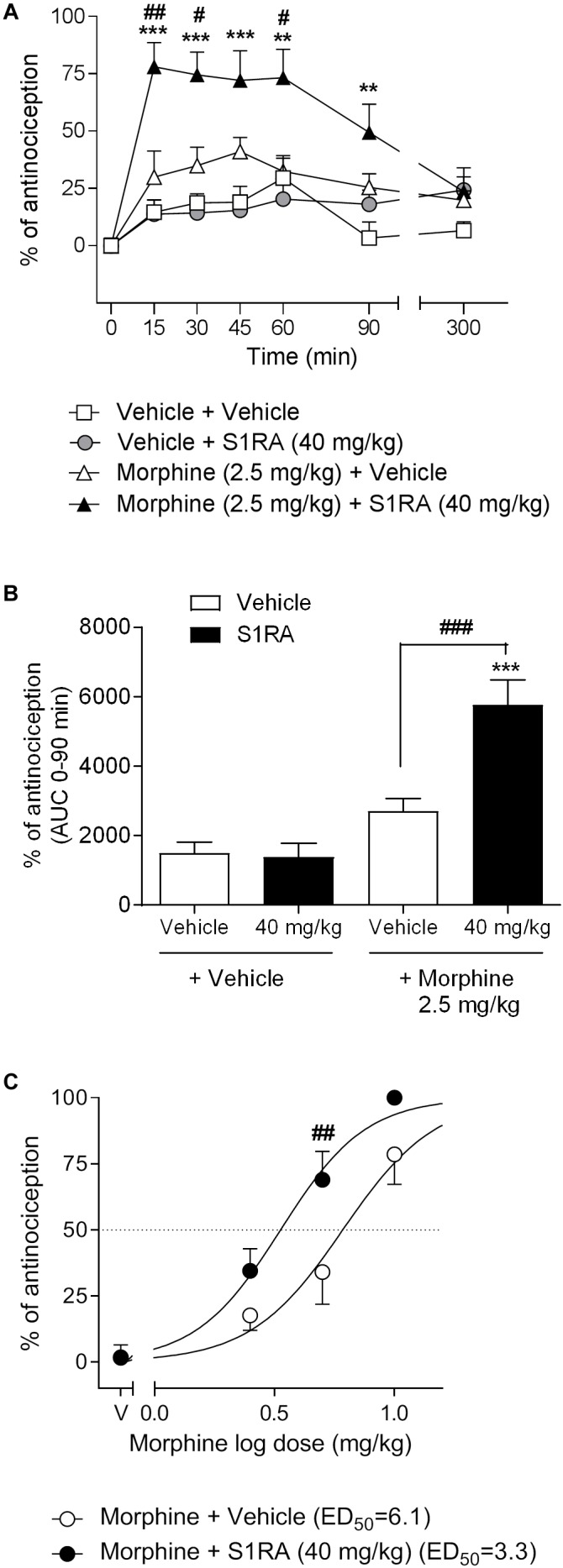
Effects of systemic co-administration of S1RA with morphine in the tail-flick test in rats. **(A)** Rats received morphine (2.5 mg/kg, i.p.), S1RA (40 mg/kg, i.p.), their combination or respective vehicles, and the tail-flick latency was evaluated over time. Note that the enhancement of the antinociceptive effect was clearly observed 15 min post-treatment and lasted up to 90 min post-treatment. Each point and vertical line represents the mean ± S.E.M. percentage of antinociception (*n* = 9–10 per group). Two-way ANOVA (time × treatment) of 0–300 min interval evaluation was performed. ^∗∗^*P <* 0.01, ^∗∗∗^*P <* 0.001 vs. respective baseline values; ^#^*P <* 0.05, ^##^*P <* 0.01 vs. morphine group (Bonferroni *post hoc* test). **(B)** AUC of 0–90 min interval evaluation. ^∗∗∗^*P <* 0.001 vs. vehicle+vehicle group; ^###^*P <* 0.001 vs. morphine+vehicle group (Newman–Keuls multiple comparison test post one-way ANOVA). **(C)** Rats received increasing doses of morphine (i.p.) or vehicle + a fixed dose of S1RA (40 mg/kg, i.p.) or vehicle, and the tail-flick latency was evaluated 30 min later. Note that S1RA increased the morphine antinociceptive effect. Each point and vertical line represents the mean ± S.E.M. percentage of antinociception (*n* = 8–10 per group). Two-way ANOVA (dose × treatment) was performed. ^##^*P <* 0.01 vs. morphine 5 mg/kg+vehicle group (Bonferroni *post hoc* test).

We first investigated the antinociceptive effects elicited by the systemic co-administration of an opioid with a σ_1_R antagonist in the tail-flick test in rats. To this purpose, tail-flick latencies were assessed over time following co-administration of morphine (2.5 mg/kg, i.p.) + S1RA (40 mg/kg, i.p.) (Figure 1A). Two-way ANOVA (time × treatment) revealed a treatment effect *F*(3, 34) = 17.37, *P* < 0.001, 0–300 min. In vehicle + vehicle treated rats, the tail-flick latencies did not change significantly from the baseline values over the entire period of time (300 min). Morphine (2.5 mg/kg, i.p.) exerted a discrete, non-significant antinociceptive effect during the first 60 min post-treatment whereas S1RA (40 mg/kg, i.p.) was devoid of antinociceptive effect at any evaluated timepoint. Co-administration of S1RA with morphine produced a significant increase in the tail-flick latency over time, with maximum effect at 15–60 min post-treatment and return to baseline 300 min post-treatment (Figure 1A). AUC analysis revealed a significant enhancement of antinociception (*P <* 0.001) in the co-treated group as compared to the morphine-treated group (Figure 1B).

To further assess the potentiation of the antinociceptive effect, we next combined different doses of morphine (2.5, 5, and 10 mg/kg, i.p.) with a fixed dose of S1RA (40 mg/kg, i.p.) and tail-flick latencies were evaluated 30 min after co-administration. The combination induced a shift to the left of the dose-response curve of morphine, resulting in an enhancement of the antinociceptive potency of the opioid by a factor of 1.8. The ED_50_’s were 6.1 (95% CI, 4.8–7.8) and 3.3 (95% CI, 2.7–4.1) mg/kg for morphine alone and morphine plus 40 mg/kg of S1RA, respectively (Figure 1C). Two-way ANOVA (dose × treatment) revealed a treatment effect *F*(1, 36) = 12.94, *P* < 0.001. The morphine dose that produced a higher enhancement by S1RA was 5 mg/kg and was therefore the first dose selected for the next set of experiments.

### S1RA and Morphine Systemically Co-administered Failed to Modify Spinal Noradrenaline (NA) Levels

We previously reported that S1RA (80 mg/kg) increased NA levels in the dorsal horn of the spinal cord and that this effect correlated well with the antinociceptive effect of S1RA in the formalin-induced pain model (Vidal-Torres et al., 2014). Here we address whether S1RA enhancement of opioid antinociception is associated with a potentiation of the increase in NA spinal levels (studied in naïve or in implanted animals, respectively).

**FIGURE 2:**
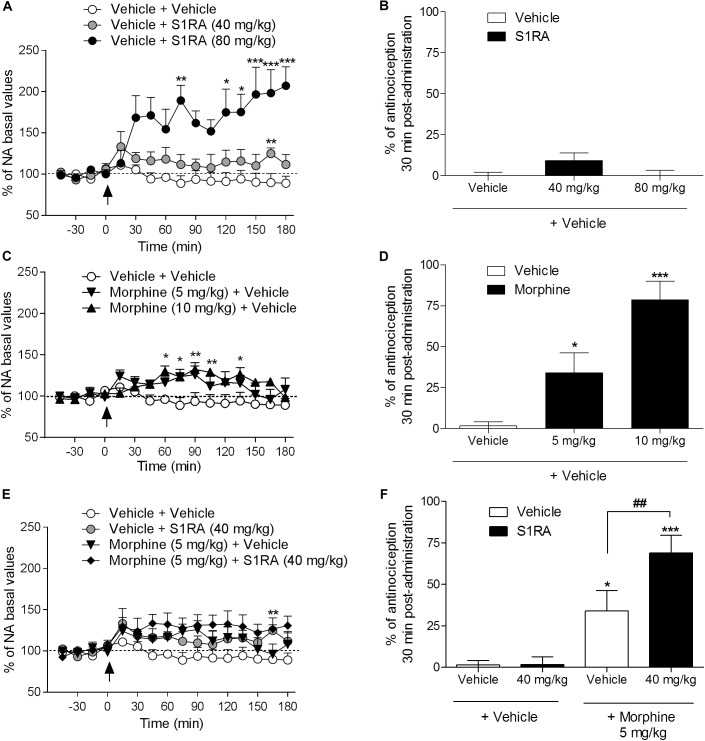
Behavioral antinociceptive effects and noradrenaline (NA) levels in the dorsal horn of the spinal cord following systemic S1RA, morphine and their combination in rats. Implanted rats received i.p. S1RA (40 and 80 mg/kg) or vehicle **(A)**, i.p. morphine (5 and 10 mg/kg) or vehicle **(C)**, or the combination of morphine (5 mg/kg) and S1RA (40 mg/kg) **(E)**, and were perfused for 180 min to evaluate the effect on extracellular concentration of NA in the dorsal horn of the spinal cord. Two-way ANOVA (time × treatment) of -45–180 min interval evaluation was performed. Dots are means ± S.E.M. values and are expressed as percentages of the respective baseline values (*n* = 4–8 per group). ^∗^*P <* 0.05, ^∗∗^*P <* 0.01, ^∗∗∗^*P <* 0.001 vs. respective baseline value (Bonferroni *post hoc* test). Naïve rats received the same treatments and 30 min later tail-flick latencies were evaluated and the percentage of antinociception elicited by treatments was calculated **(B,D,F)**. Note that S1RA at 80 mg/kg increased NA levels **(A)** but failed to produce an antinociceptive effect **(B)**. In contrast, 5 and 10 mg/kg of morphine, although they did not change NA levels 30 min post-administration **(C)**, resulted in antinociception **(D)**. The combination of S1RA (40 mg/kg) and morphine (5 mg/kg) failed to significantly modify NA values **(E)** but enhanced antinociception **(F)**. Each point and vertical line represents the mean ± S.E.M. percentage of antinociception (*n* = 8–10 per group). ^∗^*P <* 0.05, ^∗∗∗^*P <* 0.001 vs. respective vehicle+vehicle group; ^##^*P <* 0.01 vs. vehicle+morphine 5 mg/kg group (Newman–Keuls multiple comparison test post one-way ANOVA).

Two-way ANOVA (time × treatment) revealed a treatment effect *F*(2, 10) = 12.14, *P* < 0.01, -45–180 min (Figure 2A). Vehicle-treated animals showed stable NA spinal levels. NA spinal levels increased following i.p. administration of S1RA at 80 mg/kg (171% vs. baseline (100%) was found 30 min post-administration), but not at 40 mg/kg (Figure 2A). However, both doses of S1RA were devoid of antinociceptive effects at 30 min post-administration when administered alone (Figure 2B). Two-way ANOVA (time × treatment) revealed a treatment effect *F*(2, 11) = 7.84, *P* < 0.01, -45–180 min (Figure 2C). However, 30 min after i.p. administration of 5 and 10 mg/kg of morphine, NA spinal levels did not significantly differ from baseline values (114 and 115%, respectively), although significantly increased levels were attained 60 min post-administration (Figure 2C). Interestingly, both morphine doses resulted in an antinociceptive effect (*P <* 0.05 and *P <* 0.001, respectively) 30 min post-administration (Figure 2D). Two-way ANOVA (time × treatment) revealed that there was no treatment effect *F*(3, 17) = 2.68, *P* ns, -45–180 min (Figure 2E). The combination of S1RA (40 mg/kg) and morphine (5 mg/kg) enhanced antinociception (Figure 2F), but did not significantly modify extracellular NA levels vs. baseline (133%) (Figure 2E).

### Intrathecal S1RA Failed to Enhance the Antinociceptive Effect of Systemic Morphine in the Tail-Flick Test in Rats

**FIGURE 3:**
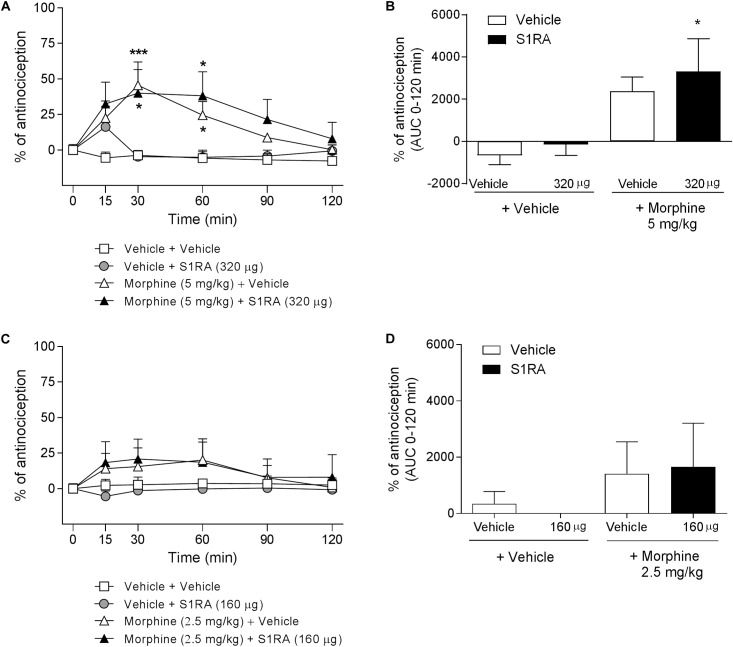
Time-related effects of intrathecal S1RA administration with systemic morphine in the tail-flick test in rats. Rats received i.p. morphine (2.5 or 5 mg/kg) or vehicle + i.t. S1RA (160 or 320 μg) or vehicle, and the tail-flick latencies were assessed over time. **(A,C)** Two-way ANOVA (time × treatment) of 0–120 min interval evaluation were performed. Each point and vertical line represents the mean ± S.E.M. percentage of antinociception (*n* = 5–10 per group). ^∗^*P <* 0.05, ^∗∗∗^*P <* 0.001 vs. respective baseline values (Bonferroni *post hoc* test). Note that morphine elicited a significant antinociceptive effect (30 and 60 min post-administration) and that this effect was not increased by i.t. S1RA. **(B,D)** AUC of 0–120 min interval evaluation. ^∗^*P <* 0.05 vs. vehicle+vehicle group; ns vs. morphine+vehicle group (Newman–Keuls multiple comparison test post one-way ANOVA).

We have previously shown that i.t. administration of 160 and 320 μg of S1RA dose-dependently reduced formalin-induced flinching but not licking/lifting behaviors. In order to investigate whether spinal σ_1_R antagonism is involved in the modulation of opioid antinociception, rats were i.t. administered with S1RA in combination with systemic morphine. Two-way ANOVA (time × treatment) revealed a treatment effect *F*(3, 31) = 3.40, *P* < 0.05, 0–120 min (Figure 3A). S1RA administered alone by i.t. route at 160 and 320 μg was inactive in the tail-flick test. Morphine (5 mg/kg, i.p.) exerted significant antinociceptive effects (*P <* 0.001) 30 min post-administration, but S1RA (320 μg) co-administered i.t. was unable to increase its analgesic effect (Figure 3A). Two-way ANOVA (time × treatment) revealed no treatment effect *F*(3, 24) = 0.81, *P* ns, 0–120 min (Figure 3C). A lower morphine dose (2.5 mg/kg) was also not enhanced by S1RA (160 μg) (Figure 3C). AUC analysis confirmed no enhancement of morphine antinociception in co-treated vs. morphine-treated groups (Figure 3B,D).

### Intracerebroventricular but Not Rostroventral Medullar S1RA Enhanced the Antinociceptive Effect of Systemic Morphine in the Tail-Flick Test in Rats

**FIGURE 4:**
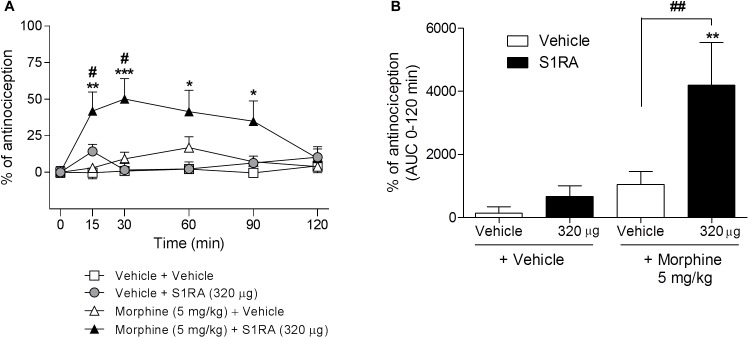
Time-related effects of intracerebroventricular S1RA administration with systemic morphine in the tail-flick test in rats. Rats received i.p. morphine (5 mg/kg) or vehicle + i.c.v. S1RA (320 μg) or vehicle, and the tail-flick latencies were evaluated over time. Note that i.c.v. S1RA increased the antinociceptive effect of systemic morphine. **(A)** Two-way ANOVA (time × treatment) of 0–120 min interval evaluation was performed. Each point and vertical line represents the mean ± S.E.M. percentage of antinociception (*n* = 7–9 per group). ^∗^*P <* 0.05, ^∗∗^*P <* 0.01, ^∗∗∗^*P <* 0.001 vs. respective baseline values; ^#^*P <* 0.05 vs. vehicle+morphine group (Bonferroni *post hoc* test). **(B)** AUC of 0–120 min interval evaluation. ^∗∗^*P <* 0.01 vs. vehicle+vehicle group; ^##^*P <* 0.01 vs. vehicle+morphine group (Newman–Keuls multiple comparison test post one-way ANOVA).

We had previously shown that 320 μg of i.c.v. S1RA significantly reduced formalin-induced pain behaviors. Here we assessed whether supraspinal σ_1_R antagonism potentiates morphine antinociception. Two-way ANOVA (time × treatment) revealed a treatment effect *F*(3, 30) = 6.05, *P* < 0.01, 0–120 min. S1RA (320 μg) administered i.c.v. and systemic morphine (5 mg/kg, i.p.) did not significantly modify tail-flick latencies in i.c.v.-implanted rats when both compounds were administered alone. However, their combination resulted in a significant enhancement (*P <* 0.05) of the antinociception at 15 and 30 min post-administration (Figure 4A). AUC analysis revealed a significant enhancement of antinociception (*P <* 0.01) in co-treated vs. morphine-treated groups (Figure 4B).

**FIGURE 5:**
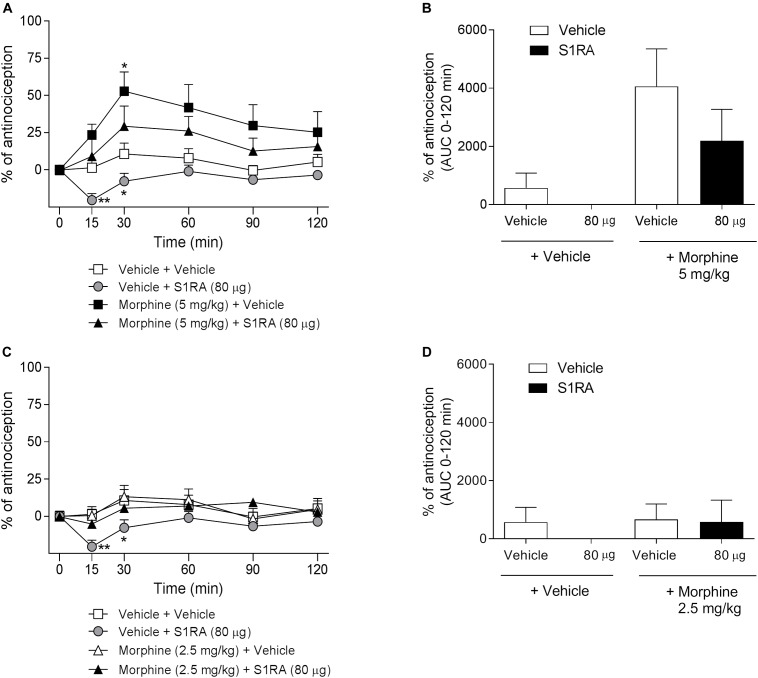
Time-related effects of rostroventral medulla S1RA administration with systemic morphine in the tail-flick test in rats. Rats received i.p. morphine (2.5 or 5 mg/kg) or vehicle + RVM S1RA (80 μg) or vehicle, and the TFL was evaluated over time. **(A,C)** Two-way ANOVA (time × treatment) of 0–120 min interval evaluation was performed. Note that morphine exhibited significant antinociceptive effects (30 min post-administration) that were not increased by RVM S1RA. Each point and vertical line represents the mean ± S.E.M. percentage of antinociception (*n* = 6–8 per group). ^∗^*P <* 0.05, ^∗∗^*P <* 0.01 vs. respective baseline values (Bonferroni *post hoc* test). **(B,D)** AUC of 0–120 min interval evaluation. ns vs. vehicle+morphine group (Newman–Keuls multiple comparison test post one-way ANOVA).

RVM was reported to be a key area for opioid modulation by some σ_1_R ligands (Mei and Pasternak, 2007). To further explore the supraspinal site for σ_1_R-mediated potentiation of opioid antinociception, we investigated the involvement of the RVM in such a potentiation. To this purpose, intra-RVM administration of S1RA (80 μg) was combined with systemic morphine (2.5 and 5 mg/kg, i.p.). Two-way ANOVA (time × treatment) revealed a treatment effect *F*(3, 23) = 4.45, *P* < 0.05, 0–120 min only in Figure 5A. RVM microinjection of S1RA (80 μg) alone exerted a significant pronociceptive effect in the tail-flick test at 15 and 30 min post-administration. Morphine at 2.5 mg/kg i.p. was devoid of effect (Figure 5C) but exhibited a significant antinociceptive effect at 30 min post-treatment when administered at 5 mg/kg i.p. (*P <* 0.05) (Figure 5A). When S1RA (80 μg, intra-RVM) and morphine (2.5 and 5 mg/kg, i.p.) were combined, no significant change vs. the effect exerted by morphine alone was observed. AUC analysis revealed no significant enhancement of antinociception in co-treated vs. morphine-treated groups (Figure 5B,D).

### Systemic S1RA Enhanced the Antinociceptive Effect of Systemic Loperamide in the Tail-Flick Test in Rats

**FIGURE 6:**
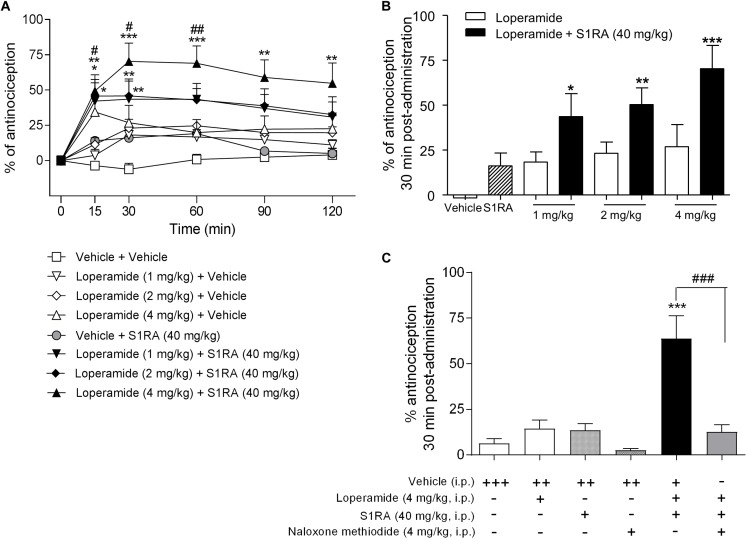
Time-related effects of systemic S1RA administration with systemic loperamide in the tail-flick test in rats. **(A)** Rats received i.p. loperamide (1, 2, and 4 mg/kg) or vehicle + i.p. S1RA (40 mg/kg) or vehicle, and the tail-flick latencies were evaluated over time. Two-way ANOVA (time × treatment) of 0–120 min interval evaluation was performed. Note that loperamide effects were enhanced by systemic S1RA. Each point and vertical line represents the mean ± S.E.M. percentage of antinociception (*n* = 6–10 per group). ^∗^*P <* 0.05, ^∗∗^*P <* 0.01, ^∗∗∗^*P <* 0.001 vs. vehicle-treated group; ^#^*P <* 0.05, ^##^*P <* 0.01 vs. corresponding loperamide dose (Bonferroni *post hoc* test). **(B)** Effects at 30 min post-administration. ^∗^*P <* 0.05, ^∗∗^*P <* 0.01, ^∗∗∗^*P <* 0.001 vs. vehicle-treated group (Newman–Keuls multiple comparison test post one-way ANOVA). **(C)** Animals were pre-treated with i.p. naloxone-methiodide (4 mg/kg) 5 min prior to i.p. loperamide (4 mg/kg) and i.p. S1RA (40 mg/kg), and evaluated at 30 min post-administration. Note that enhancement of the loperamide effect by S1RA was blocked by naloxone-methiodide. Each point and vertical line represent the mean ± S.E.M. percentage of antinociception (*n* = 8–12 per group). ^∗∗∗^*P <* 0.001 vs. vehicle (+++) group; ^###^*P <* 0.001 vs. loperamide+S1RA+vehicle group (Newman-Keuls multiple comparison test post one-way ANOVA). +, ++, +++ represents the number of administrations; - means no administration.

In order to address the involvement of σ_1_R in opioid antinociception at the periphery, different doses of the peripheral μ-opioid agonist loperamide (1, 2, and 4 mg/kg, i.p.) were co-administered with a fixed dose of S1RA (40 mg/kg, i.p.) in the tail-flick test in rats. Two-way ANOVA (time × treatment) revealed a treatment effect *F*(7, 49) = 4.90, *P* < 0.001, 0–120 min. Loperamide alone did not elicit significant antinociceptive responses but did dose-dependently elicit antinociception when combined with S1RA over time, with maximum effect observed at 30 min post-treatment (Figure 6A,B). In another set of confirmatory experiments, animals were only measured at baseline and 30 min after loperamide and S1RA co-administration, and the effect was assessed in the presence of the peripherally acting μ-opioid receptor antagonist naloxone-methiodide. Pre-treatment with naloxone-methiodide (4 mg/kg, i.p.) blocked the potentiating effect of the loperamide + S1RA combination (Figure 6C).

## Discussion

The present study demonstrated that supraspinal and peripheral, but not spinal, S1RA administration enhances opioid antinociception and that such a potentiating effect occurs without a concomitant increase in spinal NA release, in contrast to what is described for the formalin-induced pain model (Vidal-Torres et al., 2014).

The acute tail-flick response to nociceptive thermal (heat) stimulation was used to assess the potentiating effect of S1RA on opioid antinociception in rats. Systemic S1RA (40 mg/kg) had no antinociceptive effect when given alone but significantly increased the antinociceptive effect induced by morphine (2.5 mg/kg) up to 90 min post-administration. An ED_50_ ratio value of 1.8 was obtained for morphine alone and in combination with S1RA (40 mg/kg) 30 min after administration. This value was similar to that previously obtained for S1RA in mice (2.4) and for haloperidol in rats (2) (Chien and Pasternak, 1994; Vidal-Torres et al., 2013).

Because we previously argued that S1RA modulates the analgesic effect in the formalin test by increasing spinal NA levels (Vidal-Torres et al., 2014), we dialysed the dorsal horn of the spinal cord after co-administration of morphine and S1RA at doses exerting antinociceptive effects. This technique allowed us to study spinal neurochemical modulation at the dorsal horn level in awake, freely-moving rats (Vidal-Torres et al., 2012). Subactive doses of S1RA and morphine, when combined, enhanced opioid antinociception in the tail-flick test, but failed to modify NA concentrations vs. baseline. In fact, morphine induced a dose-dependent antinociceptive effect without concomitantly increasing NA spinal levels, and S1RA (80 mg/kg) *per se* increased spinal NA levels but failed to evoke antinociceptive effects in the tail-flick test. Therefore, opioid antinociception and potentiation of opioid antinociception did not correlate well with an enhancement of NA levels in the dorsal horn of the spinal cord. This fact discards the change in spinal NA levels as a key mechanism underlying opioid antinociception and σ_1_R antagonism-mediated potentiation of opioid antinociception in the spinal reflex tail-flick response to an acute thermal stimulation. This contrasts with the previous findings suggesting that increased NA levels lie behind the antinociceptive effect of S1RA in the formalin test (Vidal-Torres et al., 2014). Therefore, we might difference two S1RA-mediated mechanisms of action for analgesia depending of the spinal NA involvement. However, caution should be exerted when interpreting and extrapolating these results, as the involvement of spinal NA seems to differ depending on the nature of the painful stimuli and the outcome measure of the response. In addition, it cannot be discarded an involvement of other neurotransmitters (serotonin, endogenous opioid peptides…) in the σ_1_R antagonism on opioid analgesia in the descending pain control pathway.

The site of action of σ_1_R modulation of opioid analgesia was addressed in a few studies using non-selective sigma compounds at the supraspinal and spinal levels (Mei and Pasternak, 2002, 2007; Marrazzo et al., 2006; Tseng et al., 2011) and more recently using S1RA at the peripheral level (Tejada et al., 2017, 2018). In the present study we took advantage of using the selective σ_1_R antagonist S1RA to investigate the contribution of peripheral, spinal and supraspinal σ_1_R blockade on morphine antinociception enhancement in the tail-flick acute thermal nociceptive pain model in rats.

Firstly, we found that i.t. and i.c.v. S1RA treatment alone failed to produce antinociception in the tail-flick test at the same doses inducing clear-cut antinociceptive effects in the formalin-induced pain model (Vidal-Torres et al., 2014). These results are not surprising given that systemic S1RA by itself did not produce antinociceptive effects in the tail-flick test, and are consistent with previous studies reporting that σ_1_R antagonism elicits antinociception in sensitizing conditions but does not affect perception of normal nociceptive stimuli (e.g., perception of thermal stimulation in the tail-flick test) (de la Puente et al., 2009; Nieto et al., 2012; Romero et al., 2012). I.t. S1RA attenuated the flinching behavior (phases I and II) but not the lifting/licking response in the formalin test. These results in the formalin test can be reconciled if we consider that the lifting/licking response requires supraspinal integration, whereas the flinching behavior is essentially a spinal response that does not require the integrative action of higher brain centers. Accordingly, σ_1_R antagonists acting locally at the spinal cord level seem to modulate the spinal reflex output but not motor neuron responses integrating descending, supraspinally processed outputs. While this fits well with data in the formalin test, i.t. S1RA did not inhibit the tail withdrawal response in the tail-flick test, which is also considered to be a spinal response (Irwin, 1962). Differences in the nociceptive stimuli (thermal vs. chemical), which recruit different spinal pathways/mechanisms being differentially regulated (or not regulated at all) by σ_1_R, could provide an explanation. In this regard, i.t. administration of the σ_1_R antagonist BD-1047 is known to attenuate mechanical allodynia but not thermal hyperalgesia in a neuropathic pain model (Roh et al., 2008). Alternatively, the difference could be related to the duration of the stimulus, as thermal stimulation in the tail-flick test evokes immediate withdrawal/guarding responses whereas formalin-induced pain, even in phase I, lasts for several minutes, and thus some degree of sensitization may occur. This wider operating window gives σ_1_R antagonists, which are known to inhibit spinal wind-up sensitization phenomena (Romero et al., 2012; Mazo et al., 2015), the opportunity to exert their effect.

Secondly, our results revealed that i.c.v. but not i.t. administration of S1RA in combination with systemic morphine enhanced morphine antinociception in the co-treated group as compared to the morphine-treated group. The lack of effect of i.t. administration on opioid antinociception might be related to the poor co-expression of both targets in the same spinal cord region: the dorsal horn expresses high levels of opioids receptors but not σ_1_R which is highly expressed in the ventral horn of the spinal cord (Mavlyutov et al., 2016). These effects of i.c.v and i.t. administration of S1RA on opioid antinociception are consistent with those previously described by Mei and Pasternak in mice (Mei and Pasternak, 2002). They found diminished systemic morphine antinociception when the σ_1_R agonist (+)pentazocine was given i.c.v., but no effect of (+)pentazocine against morphine when both were given spinally. Similarly, down-regulation of supraspinal σ_1_R using an antisense approach potentiated systemic and i.c.v. morphine effects (Mei and Pasternak, 2002). The supraspinal regional localization relevant to σ_1_R-mediated modulation of opioid antinociception is only beginning to be clarified. PAG, LC, and RVM, areas where σ_1_R is expressed (Walker et al., 1992), are relevant morphine-sensitive sites (Rossi et al., 1993, 1994). Morphine antinociception was lowered by co-administration of low doses of (+)-pentazocine in all three regions (although PAG was far less sensitive than the others), thus implying a highly sensitive σ_1_ system. Only RVM seems to have a tonic σ_1_ activity based upon the ability of the σ_1_R antagonist haloperidol and the antisense treatment to enhance morphine actions (Mei and Pasternak, 2007). Nevertheless, S1RA (80 μg) administered into the RVM failed to increase the tail-flick latency when given alone and also failed to enhance the effects of systemic morphine. These results suggest that the σ_1_R system in this brainstem region (RVM) does not enhance systemic morphine antinociception in the tail-flick test. In contrast to the study by Mei and Pasternak (2007), in which morphine was microinjected together with the σ_1_R ligand, in our experiment morphine was administered systemically. In addition, S1RA when given alone into the RVM produced a slight decrease in the tail-flick latency at 15 and 30 min after the administration in our experimental conditions. Therefore, we cannot discard that the short-term pronociceptive effect of S1RA could be a reason why S1RA did not potentiate morphine antinoception. This makes this area especially interesting for further studies to understand the physiological consequences of a possible pronociceptive action of the S1RA when given in the RVM.

Finally, we showed that σ_1_R plays an important role on peripheral opioid-mediated acute thermal antinociception. We tested the effects of S1RA on the modulation of analgesia by using the peripherally acting μ-opioid agonist loperamide (Heykants et al., 1974; Schinkel et al., 1996). Loperamide (1, 2, and 4 mg/kg) was devoid of antinociceptive effects in the tail-flick in rats, in agreement with previous reports (Menéndez et al., 2005; Sevostianova et al., 2005) and consistent with the view that analgesic effects of opioids on acute pain are primarily mediated through receptors located in the central nervous system (Yaksh and Rudy, 1978; McNally, 1999). Interestingly, systemic loperamide produced a marked antinociceptive effect when combined with S1RA (40 mg/kg). The recruitment of peripheral opioid receptors in the antinociception produced by loperamide in the presence of S1RA was confirmed by its sensitivity to the reversion by the peripherally restricted opioid antagonist naloxone methiodide (Russell et al., 1982). Therefore, the tonically active anti-opioid sigma-1 system, previously described by Pasternak, works not only at central levels, but also at peripheral sites. In fact, the density of σ_1_Rs in the DRG was found to be much higher than in brainstem areas or in the dorsal spinal cord (Sánchez-Fernández et al., 2014), pointing to a prominent role for peripheral σ_1_Rs in pain modulation. In fact, the administration of a σ_1_R antagonist was sufficient to unmask the opioid effect of loperamide, a peripherally restricted mu opioid agonist commonly used as antidiarrheal drug. The molecular mechanism of the interaction between σ_1_Rs and the mu opioid receptor was recently elucidated. Sigma-1 antagonism increases mu-opioid signaling through a complex regulation of the interaction between NMDA receptors and mu-opioid receptors, two of the main protein targets of σ_1_Rs (Rodríguez-Muñoz et al., 2015). Although the specific role of σ_1_R on pain modulation at the periphery has not been extensively studied, our results are in agreement with those recently reported by Cobos and coworkers, where S1RA and other σ_1_R antagonists did not modify nociceptive thresholds when administered locally at the periphery (intraplantarly) but did potentiate opioid mechanical antinociception. Interestingly, the sigma-1 tonic inhibitory actions on peripheral opioid seem to be limited to the mechanical stimuli because σ_1_R inhibition did not potentiate other peripherally-mediated opioid effects, such as constipation, or peripheral opioid antinociception to heat stimuli (Sánchez-Fernández et al., 2014, 2017; Montilla-García et al., 2018). It has also been described that σ_1_R antagonists alone exert remarkable antinociceptive effects at the periphery in conditions involving inflammation by modulating the analgesic effects of endogenous opioid produced by immune cells at the periphery (Tejada et al., 2017, 2018). Interestingly, loperamide alone produces peripheral analgesia also in inflammatory pain conditions (Khalefa et al., 2012). Altogether, the antagonism on σ_1_R at the periphery may be used as a local adjuvant strategy to enhance peripheral μ-opioid analgesia while avoiding the undesirable central opioid-mediated side effects, thus increasing the opioid benefit-to-risk ratio.

## Conclusion

In conclusion, the studies herein suggest that the σ_1_R antagonism enhances opioid antinociception in acute thermal pain conditions by the sum/integration of supraspinal and peripheral effects, through a mechanism independent of spinal NA levels.

## Ethics Statement

All animal husbandry and experimental procedures complied with the European guidelines for the protection of animals used for experimental and other scientific purposes (Council Directive of 22 September 2010, 2010/63/EU), and were approved by the local Ethics Committee.

## Author Contributions

AV-T, BF-P, and DZ designed and conducted the research. AV-T, AC, and BF-P performed the experiments. AV-T, JV, MM, and DZ wrote the main manuscript text. All authors analyzed the results and reviewed the manuscript.

## Conflict of Interest Statement

The authors of this article are employees of Esteve Pharmaceuticals.
